# BioChainReward: A Secure and Incentivised Blockchain Framework for Biomedical Data Sharing

**DOI:** 10.3390/ijerph20196825

**Published:** 2023-09-25

**Authors:** Mahmoud Elkhodr, Ergun Gide, Omar Darwish, Shorouq Al-Eidi

**Affiliations:** 1 School of Engineering and Technology, Central Queensland University, Sydney, NSW 2000, Australia; ergun.gide1@cqu.edu.au; 2 Information Security and Applied Computing Department, Eastern Michigan University, Ypsilanti, MI 48197, USA; odarwish@emich.edu; 3 Computer Science Department, Tafila Technical University, Tafila 66110, Jordan; shorouqa@mun.ca

**Keywords:** biomedical data, blockchain, EHR, health informatics, privacy security

## Abstract

In the era of digital healthcare, biomedical data sharing is of paramount importance for the advancement of research and personalised healthcare. However, sharing such data while preserving user privacy and ensuring data security poses significant challenges. This paper introduces BioChainReward (BCR), a blockchain-based framework designed to address these concerns. BCR offers enhanced security, privacy, and incentivisation for data sharing in biomedical applications. Its architecture consists of four distinct layers: data, blockchain, smart contract, and application. The data layer handles the encryption and decryption of data, while the blockchain layer manages data hashing and retrieval. The smart contract layer includes an AI-enabled privacy-preservation sublayer that dynamically selects an appropriate privacy technique, tailored to the nature and purpose of each data request. This layer also features a feedback and incentive mechanism that incentivises patients to share their data by offering rewards. Lastly, the application layer serves as an interface for diverse applications, such as AI-enabled apps and data analysis tools, to access and utilise the shared data. Hence, BCR presents a robust, comprehensive approach to secure, privacy-aware, and incentivised data sharing in the biomedical domain.

## 1. Introduction

As healthcare systems become increasingly digital, ensuring the secure and efficient exchange of sensitive patient data is pivotal. Thus, storing and sharing electronic health records (EHRs), including voluminous biomedical data, such as telehealth videos, medical images, and genomic sequences, pose a significant challenge in terms of security and privacy. Medical imaging data, being high-dimensional and uniquely identifying, can be exploited for malicious intent if not handled securely, leading to privacy infringements [1]. Genomic sequences, carrying deep and personal biological information, could lead to profound privacy violations and ethical issues if not appropriately shared and secured [2]. Traditional centralised database solutions run the risk of data breaches, loss, and misuse and incur high storage and bandwidth costs. Cloud computing-based solutions, such as the framework proposed in [3], offer some advantages, such as scalability, flexibility, and cost-effectiveness. However, cloud-based solutions also lack sufficient guarantees for privacy and trust. In the healthcare context, centralised database solutions, while providing efficiency, struggle with key security risks. These systems lack comprehensive security measures and are prone to single-point failures and security attacks, leading to potential data breaches and patient privacy concerns [4]. Cryptography, though it secures data confidentiality, lacks in transparency and comprehensive auditability. Therefore, the significant associated privacy and security concerns challenge the widespread adoption of EHR systems. Unauthorised access, modification, or disclosure of sensitive information is a risk when biomedical data are shared among different parties. Therefore, robust security and privacy measures are needed to safeguard biomedical data’s confidentiality, integrity, authenticity, and availability. Cryptography, which uses mathematical methods to encrypt data, offers a fundamental technique for such security measures. Despite the proposal of several cryptography-based solutions to address EHR privacy and security issues, ensuring comprehensive security for sharing EHRs and biomedical data remains an ongoing challenge. Cryptography, while critical in tackling this challenge, must form part of a more prominent solution. In contrast, blockchain technology offers unique advantages specifically beneficial for healthcare data sharing. Its decentralisation creates a network of trust among untrusted parties without a central authority, enhancing security and privacy for sharing sensitive healthcare data [5]. Moreover, its transparency allows any network participant to verify each transaction, ensuring the integrity of shared EHRs [6]. The inherent immutability in blockchain, implying that once a transaction is recorded it cannot be altered, provides a robust mechanism to preserve the authenticity of biomedical data [5]. Therefore, blockchain offers tremendous opportunities in healthcare where sensitive patient data must be securely stored and shared. Thus, blockchain can overcome the challenges encountered in healthcare digital systems by enabling distributed storage of large volumes of data on disparate off-chain systems and providing a secure encrypted mechanism for patients to share access to their data, regardless of their digital location [7].

Previous studies have proposed several blockchain-based solutions for various healthcare applications, such as secure storage, access, and data provenance [8]. For example, blockchain can enhance healthcare systems’ security by generating a unique hash value for each data item and embedding it into the blockchain transaction, thereby verifying data authenticity and integrity without compromising system and data security [9,10]. Through smart contracts, blockchain can also verify and enforce data owners’ policies and preferences for data sharing, such as data access, conditions, and duration. Furthermore, blockchain can maintain the provenance and consent information of the data by generating an immutable and auditable record of all data transactions performed on the blockchain network, thus facilitating traceability, accountability, and compliance [9,10,11].

Our previous work [12] introduced a framework that securely authenticates patients and provides a secure way to share biomedical data using cloud computing technologies. Further enhancements to the framework were presented in [13], which incorporated blockchain into the framework. This current work builds on those initiatives and introduces BioChainReward (BCR), a blockchain-based framework for secure and privacy-preserving biomedical data sharing. BCR distinguishes itself from previous iterations by implementing an AI-enabled privacy-preservation sublayer, which adapts privacy measures based on the nature and purpose of the data request. Additionally, BCR introduces an incentive mechanism, rewarding patients for their data sharing, thus adding an additional aspect of user engagement to the framework. Hence, BCR represents a substantial enhancement to the biomedical data-sharing efforts introduced earlier.

This paper is organised as follows: Section 2 reviews related work; Section 3 presents the architecture and modules of the BCR framework; Section 4 evaluates the proposed framework by comparing it with existing biomedical data-sharing approaches; Section 5 discusses the limitations of this work; and Section 6 concludes the paper and suggests future directions.

## 2. Related Work

Electronic health records (EHRs) often contain sensitive biomedical data and other valuable information derived from patients’ care, treatment, prevention plans, and clinical trials. However, challenges such as data fragmentation, privacy breaches, unauthorised access, and a lack of incentives for data sharing remain. Blockchain technology offers a solution to these challenges by providing a decentralised, tamper-proof, and traceable platform for storing and managing data. Several blockchain-based schemes have been proposed to secure data sharing while preserving patient privacy. For instance, Zou et al. [14] proposed a public blockchain-based eHealth system, SPChain, which uses block structures for quick data retrieval and proxy re-encryption schemes for secure data sharing. A systematic review of 65 blockchain-based solutions for EHRs was reported in [15]. The study identified 14 main challenges that need to be addressed. It also proposed EHRChain, a dual-blockchain framework based on Hyperledger Sawtooth and IPFS that aims to tackle all the challenges simultaneously. The study reported in [16] proposed a modular system that protects patient’s privacy and data in the healthcare system by integrating two existing access control models (RBAC and ABAC) and applying pseudonymisation and anonymisation techniques.

Other studies have presented various approaches and frameworks, each providing unique benefits and facing its limitations and challenges [17,18,19,20]. Despite these promising efforts, these studies have left certain challenges unaddressed. For instance, SPChain [14], while effective for quick data retrieval and secure sharing, lacks a mechanism to incentivise patients for data sharing, which could hinder wider adoption. Similarly, although frameworks such as BlockMedCare [21], HealthBlock [22], and Medchain [23] offer novel solutions for data integrity and access control, their scope does not extend to accommodate AI-driven privacy adaptations, causing potential limitations in complex data sharing scenarios.

In addition to these studies, blockchain technology has been applied in other aspects of Internet of Things (IoT)-based healthcare, such as real-time data sharing, electricity trading, drug supply chain management, and clinical trials. Ref. [24] provided an overview of blockchain applications in healthcare and the IoT, highlighting the benefits and challenges of this technology. Ref. [25] reviewed the use of blockchain for IoT-based healthcare, focusing on the background, consensus mechanisms, platforms, and use cases. They also discussed the open issues and future directions of this research area. Ref. [10] analysed the potential of blockchain technology to provide innovative solutions in various sectors, including healthcare. They identified and discussed significant applications of blockchain for healthcare, such as in data storage, clinical trials, and insurance. Ref. [26] proposed a blockchain-based framework for smart city applications, where IoT devices can share data securely and efficiently. They demonstrated the feasibility of their framework using a case study of electricity trading among smart homes.

Several other studies have proposed blockchain-based solutions to ensure data confidentiality, integrity, and access control. For example, the authors of [21] presented BlockMedCare, a system that integrates the IoT, Ethereum-based blockchain, and the InterPlanetary File System. Ref. [22] introduced HealthBlock, a decentralised healthcare management system. Ref. [23] developed Medchain, a data-sharing platform for electronic health records and physiological data from Internet of Medical Things (IoMT) devices. However, Medchain has limitations in availability and scalability. To address the challenges of privacy leakage and data tampering by unauthorised users, the authors of [27] proposed an attribute-based secure access control mechanism that uses federated deep learning (FDL). Moreover, various biometric-based authentication systems have been explored to secure IoT-based healthcare systems [12,28,29].

Persistent challenges, like scalability, interoperability, and usability issues, necessitate the development of more efficient consensus mechanisms and novel solutions integrating blockchain with other emerging technologies. Addressing the security, lack of trust, and lack of motive aspects of sharing EHRs and designing scalable and efficient applications are also areas requiring further research. To this end, this work contributes to this growing field by proposing the BioChainReward framework.

## 3. BioChainReward: A Blockchain-Based Framework for Secure and Transparent Biomedical Data Sharing

This section presents the BCR framework. It includes a section introducing the BCR architecture, followed by a section providing a use case study and another demonstrating the applicability of the BCR to this study. This is followed by two sections: one dedicated to the feedback and incentive mechanism and the other focusing on the AI-enabled privacy preservation layer.

Biomedical data contained in EHRs can significantly enhance healthcare quality and outcomes through data mining and analysis and facilitate new disease discoveries or early intervention plans, fostering medical research and innovation. However, managing EHRs presents several challenges due to the sensitive and personal nature of these data, hence necessitating a framework that enables secure, transparent biomedical data sharing among various stakeholders while preserving patient privacy.

A previous work proposed a novel biometric-based authentication framework designed to secure Internet of Things (IoT) devices used in health monitoring [12]. This framework is comprised of two main layers:

- Sensor layer: In this layer, ECG and PPG signals are collected from a patient using wearable devices, such as a smartwatch. These signals are used to generate a unique key pair, including a public key and a private biometric key;
- Cloud layer: This layer is responsible for storing the patient’s health data, which can be accessed by authorised healthcare providers. The data are encrypted and can only be decrypted using the patient’s private biometric key.

The framework also utilises a novel encryption technique based on fully homomorphic encryption (FHE), allowing computations with encrypted data without needing to decrypt it. However, this framework does not incorporate a privacy-preservation mechanism and depends on cloud computing for the security of the framework and the patient’s data.

Subsequent advancements were made, as documented in [13]. This development redesigned the framework by incorporating blockchain technology, acknowledging the emerging need for enhanced data privacy and secure access management in the healthcare sector. It also introduced a privacy sublayer.

Building upon these earlier initiatives, the proposed BioChainReward (BCR) framework aims to develop a more patient-centric security framework for IoT healthcare applications. BCR enhances the existing approaches by integrating biometric authentication, AI-enabled blockchain technology, privacy-preservation techniques, and an incentive mechanism, thereby establishing a more comprehensive and secure framework.

### 3.1. Architecture

This section describes the architecture of the BCR framework, highlighting the various layers and modules and their corresponding benefits and challenges.

**Figure 1:**
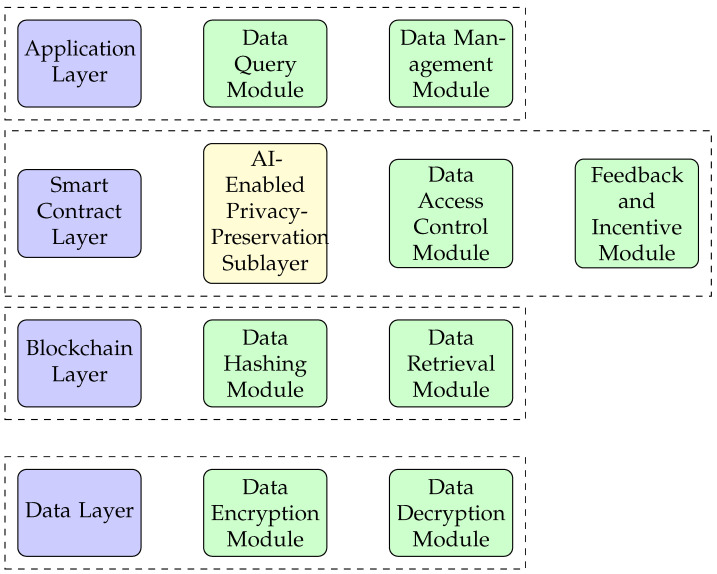
Framework architecture for BioChainReward.

As shown in Figure 1, the architecture of BCR is based on four primary layers: the data layer, the blockchain layer, the smart contract layer, and the application layer.

The data layer is responsible for the acquisition and preprocessing of patient data. It ensures the security of data through encryption before its transmission to the blockchain layer. This layer comprises the data encryption module, which encrypts data received from patients’ wearable or IoT devices, and the data decryption module, which decrypts incoming communications from healthcare professionals and configuration messages sent to IoT devices.

The blockchain layer introduces blockchain into the communication, aiming to provide immutable and transparent functionality. It comprises the data hashing module, which generates unique hash values for each data item, and the data retrieval module, which retrieves encrypted data and their associated metadata from blockchain transactions.

The smart contract layer enables data owners to control access to their data and manage their preferences for data sharing. This layer includes the data access control module and the feedback and incentive module. It also houses an artificial intelligence (AI) enabled Privacy-preservation sublayer. This sublayer utilises AI to intelligently determine and apply the most appropriate privacy technique (anonymisation, obfuscation, or differential privacy) based on the specifics of the data request. This AI-driven approach enhances the efficiency of privacy preservation and optimises security measures based on the data request’s context.

The application layer serves as the user interface that interacts with the underlying layers. This layer includes the data query module, which allows data requesters to search for and request specific data from the blockchain layer, and the data management module, which enables data owners to register and update their data and policies with the blockchain layer and the smart contract layer.

### 3.2. Use Case Study

To better illustrate the interactions between the components of the BioChainReward framework, a use-case scenario involving three main actors is presented: the first actor represents the data owner Alice (a patient), the second actor represents the requesters of data (Bob; e.g., representing a researcher), and the last actor is Charlie (representing a regulator).

#### 3.2.1. Scenario

Alice is a patient who suffers from diabetes and hypertension. She uses an IoT wearable device to monitor her blood glucose and blood pressure levels. The data collected by the IoT device are sent by the IoT gateway to a healthcare provider’s server. The data are then used by healthcare professionals to provide treatment and intervention plans for Alice. The healthcare providers also participate in research investigating the use of machine learning models for the prediction and prevention of cardiovascular diseases. They do so by granting Bob, a researcher, access to the biomedical data of Alice and other patients. Bob also has access to other data from various sources and patients, which helps him train and test his machine learning (ML) model. To assure the ethical and responsible use of the biomedical data and the sharing process, a regulator, Charlie, oversees this process.

#### 3.2.2. Applying BioChainReward to the Scenario

Alice registers her data on the blockchain layer using the data management module on her mobile app. She also specifies her data-sharing policies and preferences on the smart contract layer, including the purpose, duration, and compensation for the data sharing. These policies are then utilised by the AI-enabled privacy-preservation sublayer to enforce appropriate privacy-preserving techniques depending on the nature of the data request. There are default policies automatically checked for her in the app to simplify the process. Alice can, at any time, update or revoke specific policies or preferences from the app.

Bob uses a Web portal application to access the biomedical data of the patients participating in the research. Upon logging into the Web portal, Bob provides his authorisation credentials and details of his research project and specifies the data request; i.e., requesting Alice’s data. The framework queries Alice’s data using the data query module. The data query module sends Bob’s request to the smart contract layer where the data access control module and the AI-enabled privacy-preservation sublayer are triggered. The data access control module verifies Bob’s credentials and checks Alice’s policies and preferences for data sharing, and if access is granted, the privacy-preservation sublayer selects the appropriate privacy technique (anonymisation, obfuscation, or differential privacy).

If the request is approved, the data access control module executes the smart contract and grants Bob access to the encrypted data and their metadata on the blockchain layer. The encrypted data and their metadata are then retrieved from the blockchain layer and decrypted using the data decryption module. The decrypted data are then securely sent back to the application layer, specifically to the data query module, along with any associated provenance and consent information.

Charlie, in his capacity as a regulator, can verify and audit data transactions on the blockchain layer using his Web portal. Charlie can also monitor and enforce the ethical and legal compliance of biomedical data sharing and research using the smart contract layer.

**Figure 2:**
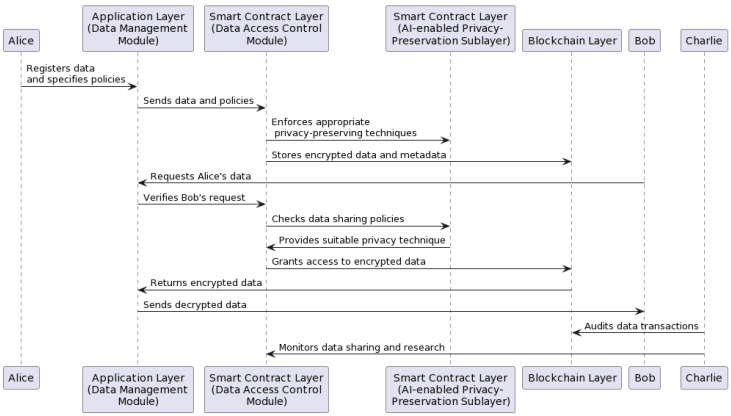
Sequence diagram.

**Figure 3:**
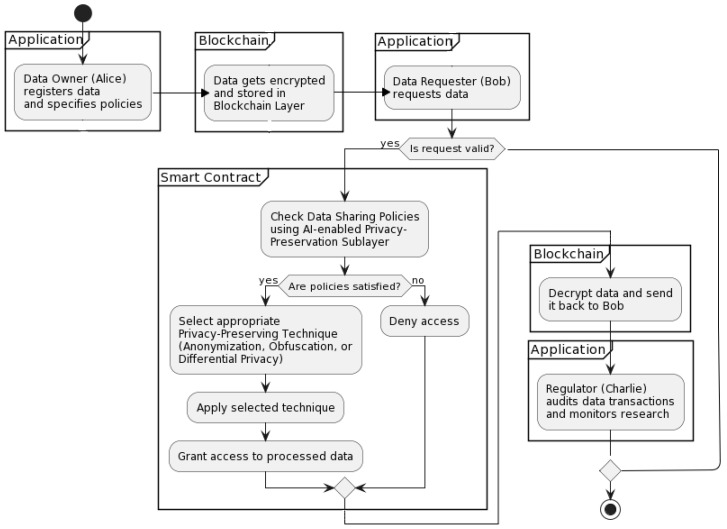
A diagram depicting the interactions within BioChainReward.

Figure 2 and Figure 3 outline the interactions between these actors in a sequence and activity diagram, demonstrating the steps and messages involved in this use case scenario, as well as the roles and interactions of the different components of the proposed BCR framework, including the AI-enabled privacy-preservation sublayer.

**Figure 4:**
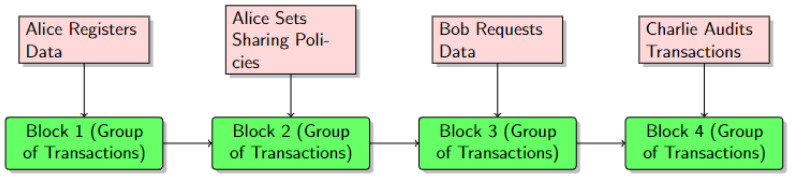
Blockchain interaction diagram.

A blockchain interaction diagram is also provided in Figure 4. It provides a detailed overview of the flow of users’ triggered events within the blockchain network. These are the transactions initiated by the users of BCR. The sequence of blocks (i.e., block one, block two, block three, and block four) is depicted in Figure 4. Each of these blocks encapsulates a group of transactions. For instance, block one represents the group of transactions in which Alice registers her data using the data module of her mobile application. For this transaction to be executed on the blockchain network, block one is created. Block two represents the group of transactions in which Alice sets her data-sharing policies and preferences using the smart contract layer. This transaction, like the first one, is added to the blockchain network, and block two is linked to block one, maintaining the chain’s integrity. Block three represents the transactions in which Bob requests access to Alice’s data using the data query module. Once Bob’s request is approved, this transaction is also added to the blockchain, creating block three, which is linked to block two. Lastly, block four contains the transactions relevant to Charlie, such as auditing the data, which are also recorded on the blockchain as block four. A link to block three is then established.

### 3.3. The Feedback and Incentive Mechanism

The framework features a feedback and incentive mechanism that rewards data owners for sharing their data and motivates them to provide high-quality and accurate data. This mechanism employs smart contracts to issue tokens or credits to data owners based on the quantity and quality of their data and the satisfaction and ratings of data requesters. The quality of data refers to the degree to which it aligns with the standardised health metrics. For instance, health data with consistent monitoring intervals, verified sources, and in line with standard health parameters would be rated as high-quality data. Data owners can use these tokens or credits to access other services or benefits in the framework, such as premium healthcare or discounts. The feedback and incentive mechanism also gathers and displays feedback and ratings from data requesters and data owners, enhancing the trust and reputation of the platform. In addition to this, if a data owner receives consistently poor feedback regarding the quality of the data, corrective action will be suggested. For example, if a patient’s health data are frequently logged at irregular intervals, resulting in incomplete datasets, the framework could suggest regular logging or automated logging through linked devices. This would ensure data consistency, thus improving the quality and accuracy of the data within the framework.

**Figure 5:**
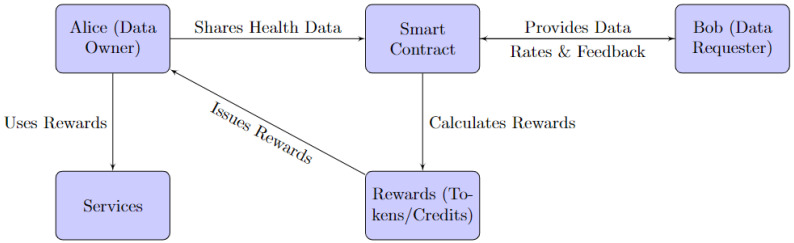
Feedback and Incentive mechanism interaction diagram.

The feedback and incentive mechanism diagram depicted in Figure 5 represents the interactive process that drives data sharing and quality in the blockchain-based framework. Alice, the data owner (in this case, a patient), shares her health data, which include her monitored blood glucose and blood pressure levels. This data are sent to the smart contract module within the blockchain layer of BCR. Alice’s health data are shared with Bob, the data requester in this scenario. As part of this interactive process, Bob provides feedback and ratings for the quality and accuracy of Alice’s data. This feedback is communicated back to the smart contract module, which is responsible for maintaining a fair and transparent data-sharing environment. The smart contract then calculates the rewards for Alice based on the quality and quantity of her shared data and the satisfaction level derived from Bob’s feedback. These rewards are issued in the form of tokens or credits. Alice can then use these tokens or credits to access various services. These services could be within the blockchain platform, such as premium healthcare features, or potentially external benefits. This incentive mechanism rewards Alice for her data contribution and encourages her to continue providing high-quality and accurate data. In this scenario, Charlie can oversee the whole process to ensure all parties are following ethical practices.

### 3.4. AI-Enabled Privacy Preservation

The BCR framework emphasises the importance of privacy-preserving techniques in ensuring data owners’ privacy rights are respected during data transactions. A significant component of this approach is the AI-enabled privacy-preservation sublayer, which automatically manages the selection and application of these techniques.

The privacy-preservation sublayer incorporates various privacy techniques, including anonymisation, obfuscation, and differential privacy. Each of these methods offers different advantages and use cases, ensuring a tailored level of security for each data transaction.

To clarify the role of anonymisation in the context of our AI-enabled privacy preservation, the AI sublayer leverages this method when the purpose of the data request does not necessitate any personally identifiable information (PII). For example, research involving population health metrics, where individual identities do not contribute to the findings, can leverage anonymised data. Through this, the sublayer ensures privacy-preserving data sharing while maintaining the usefulness of the data for broad research purposes.

Obfuscation, on the other hand, is applied when only specific data attributes are required. It involves the addition of a certain degree of noise or imprecision to the data to protect sensitive attributes while maintaining the overall utility of the data. Differential privacy is utilised when statistical summaries are required, while individual privacy is maintained. It involves adding a certain amount of random noise to the query responses to provide a level of privacy that prevents an adversary from determining whether a specific individual is present in the dataset.

Depending on the nature and purpose of the data request, the AI system within the privacy-preservation sublayer selects the most suitable method. The AI techniques employed within the privacy-preservation sublayer are based on machine learning algorithms; more specifically, decision trees. The AI system employs a decision-making process to determine the most appropriate privacy-preserving technique based on the purpose of the data request, the sensitivity level of the data, and the data owner’s privacy preferences. The AI system continually learns from the feedback received from data owners and requesters, which enhances its decision-making accuracy over time. Furthermore, robust measures, such as cross-validation techniques, are incorporated to improve the AI’s performance, ensuring a reliable privacy-preserving mechanism. It reviews the data owner’s privacy policies and preferences, applies the chosen technique to the data, and then ensures the data are encrypted and stored in the blockchain layer.

While these processes are automated and efficient, they are not hidden from scrutiny. All operations are transparent and recorded on the blockchain, allowing Charlie, in his role as a regulator, to monitor and audit these processes. This ensures that the privacy-preserving mechanisms are executed in accordance with established policies and ethical standards, further enhancing trust in the BCR framework.

**Figure 6:**
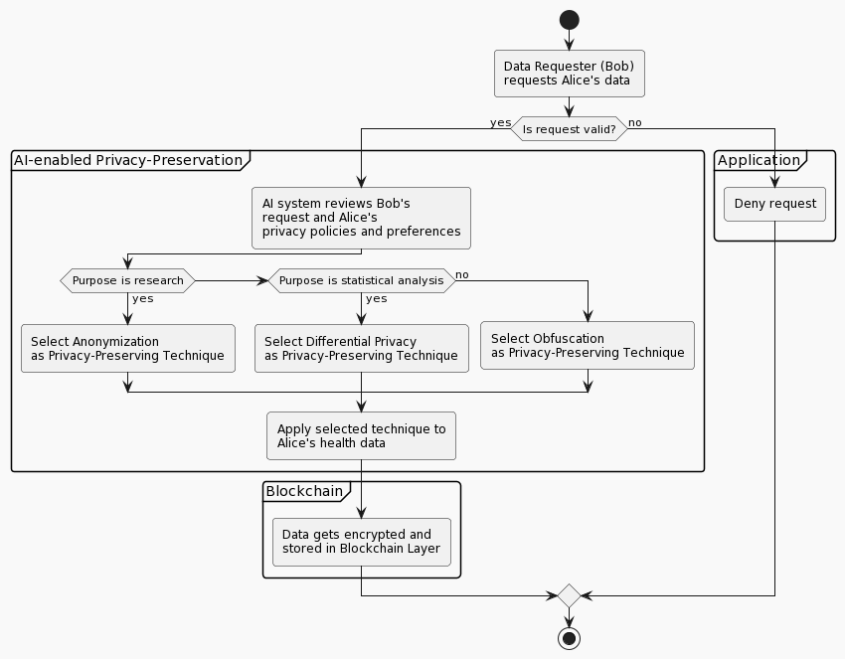
AI-enabled privacy preservation interaction diagram.

Figure 6 illustrates the sequence of operations in the privacy-preservation sublayer. It starts with Bob’s data request. Based on the purpose of his request (research, statistical analysis, or other), the AI-enabled privacy-preservation sublayer selects and applies the most appropriate privacy-preserving technique (anonymisation, differential privacy, or obfuscation) to Alice’s health data. These processed data are then encrypted and stored in the blockchain layer. Meanwhile, the regulator (Charlie) monitors and audits these privacy-preserving operations. If Bob’s request is deemed invalid, then the request is denied.

## 4. Comparing BioChainReward with Other Data-Sharing Approaches

This section presents a comparative analysis of BCR and other data-sharing approaches (namely, centralised databases, federated databases, and conventional data-sharing platforms) using a set of key metrics, as shown in Table 1. To evaluate the proposed framework, we primarily considered metrics that capture the characteristics of a robust and efficient data-sharing system. These metrics provide a comprehensive evaluation, covering aspects such as computational cost, scalability, transparency, user control, and incentive mechanisms, offering a holistic perspective on the strengths and weaknesses of the data-sharing approaches.

BCR showcases several advantages over its counterparts. By employing blockchain technology, BCR provides a decentralised, tamper-resistant framework that significantly enhances security. Scalability in BCR refers to the framework’s ability to handle an increase in data volume and the number of transactions or users. This capability is important in the context of biomedical data-sharing platforms, where the volume of data and the number of users can grow rapidly.

In the BCR framework, each participating node has the capability to process transactions independently, thereby enabling the system to handle increased data volume without becoming overwhelmed. Furthermore, the linear addition of blocks as the number of transactions grows enables the system to maintain its performance even as it scales up. In addition to these inherent features, BCR also has the potential to leverage cloud computing for data storage, thus allowing scalable and efficient data storage and retrieval. While cloud computing integration is not a part of the current framework architecture, its potential inclusion represents a significant opportunity for scalability improvement in future iterations of the framework.

In this regard, BCR, with its decentralised structure and the potential to integrate cloud storage, can handle larger volumes of data more effectively than traditional centralised or federated systems.

Moreover, BCR guarantees a higher degree of data-owner control and privacy preservation, allowing data owners to securely manage their data autonomously. The proposed BCR framework protects patients’ privacy by applying several measures throughout the data lifecycle. At the source point, security and privacy are assured using encryption. All data are encrypted before sending them to the blockchain layer, thus preventing the unauthorised use of or access to data. Importantly, only the encrypted data and their metadata are kept in the blockchain layer. The original data are stored off-chain in the data layer; e.g., using cloud computing (CC). This improves the efficiency and scalability of BCR, as the use of CC reduces the bandwidth and storage requirements of the blockchain network, thus, improving the overall performance of the framework.

Additionally, by leveraging the capability of blockchain, data integrity and authenticity are assured. Privacy is also assured by verifying and enforcing the data owners’ policies and preferences for data sharing using the data access control module in the smart contract layer. This module provides data owners with a mechanism that allows them to control access to their data by specifying the rules and conditions of access. The provenance of this interaction is captured by the smart contract layer, which stores the users’ policies and consent preferences. It also records the consent granted to data requesters and allows the users to manage and revoke this consent. Therefore, the provenance and consent information of the data are preserved through the blockchain and the smart contract layers. This enables the traceability and accountability of the data transactions and facilitates auditing and compliance. In terms of interoperability, BCR is on par with other approaches, supporting seamless data sharing among different systems. Trust and reputation are reinforced in BCR by eliminating intermediaries, reducing risks of tampering, and providing a transparent audit trail. Furthermore, BCR introduces an incentive mechanism that encourages data sharing among users, fostering a collaborative environment.

In contrast, centralised databases store biomedical data in a single location, simplifying data management but also producing single points of failure and vulnerability to cyber-attacks [6]. Data owners often have limited control over their data in such systems, raising privacy concerns. Federated databases distribute data across multiple nodes but maintain some centralisation for data management, lacking complete transparency and autonomy for data owners [7]. Conventional data sharing platforms generally depend on intermediaries like data brokers, introducing trust issues, additional costs, and delays in the data sharing process [8].

In terms of delay, BCR ensures quicker access to requested data due to the absence of intermediaries and efficient processing mechanisms, reducing time delays significantly compared to other systems.

Overall, the analysis highlights the improvement in performance with BCR compared to other data-sharing approaches in key metrics. BCR outperforms the other approaches in critical areas, such as security, data-owner control, privacy preservation, trust and reputation, incentive mechanisms, and time delay, reflecting the distinct benefits of its decentralised, blockchain-based design. While centralised databases offer low computational complexity and cost, their limited scope in terms of security, data-owner control, transparency, and privacy preservation indicates significant shortcomings in these areas. Federated databases and conventional data-sharing platforms demonstrate improved scalability and interoperability. Nonetheless, they fall short in areas such as data-owner control, privacy preservation, and incentive mechanisms.

By providing a comprehensive and comparative analysis of different data-sharing approaches, this study underscores the potential and advantages of the BCR framework in promoting secure, scalable, and efficient biomedical data sharing.

## 5. Limitations

Despite the promising implications of BCR for secure, private, and incentivised data sharing, there are several limitations and areas for future research.

Firstly, the framework proposed in this paper is a high-level architecture, and more detailed studies are required to explore the specific cryptographic solutions used in the data encryption and data decryption modules within the application layer. Lightweight cryptographic algorithms could be a beneficial avenue to explore in future research to evaluate their efficiency and impact on the overall system performance.

Secondly, the complexity of user control policies for non-expert users suggests a need for further research on the user interface (UI) and user experience (UX) of the data access control module in the smart contract layer. Emerging technologies, such as artificial intelligence (AI) or agent-based software, could potentially augment the functionality of this layer.

Thirdly, the feedback incentive mechanism’s reliance on quality ratings from researchers introduces the possibility of personal bias influencing the rating process. Future research could explore mechanisms to automate this process or create a uniformly defined rating and reward system to limit the impact of individual bias. This could also involve establishing mechanisms for dispute resolution and mitigation.

The feedback incentive mechanism also raises potential ethical and legal concerns. Incentivising patients to share their data, particularly sensitive health data, involves careful consideration of consent, privacy, potential exploitation, and potential violation of laws and regulations. While BCR provides measures to protect user privacy and offers users control over their data, more detailed exploration of these ethical and legal implications is necessary. Furthermore, future work should investigate how BCR can address these concerns while maintaining its objective of promoting data sharing. More specifically, the token-based incentive system of BCR is a key component of the framework and designed to motivate users to share their data. However, it also introduces potential concerns that warrant further research. Notably, the efficacy of the token-based incentive system in motivating data sharing remains an empirical question. The ways in which users value and respond to such incentives can vary widely and depend on numerous factors. Therefore, future research should investigate the extent to which this token/reward system can effectively incentivise data sharing and what factors may influence its success.

Moreover, there are potential risks associated with encouraging users to disclose their sensitive data for rewards. Users might be tempted to disclose more data than they otherwise would without fully understanding the privacy implications of doing so. As such, it is crucial to develop strategies to ensure that users are fully informed about the potential risks and rewards of data sharing. Future research should explore possible incentive technologies and strategies, as well as methods to clearly communicate the privacy implications to users.

While the BCR’s token/reward system presents an innovative approach to incentivising data sharing, there are numerous aspects that require further investigation. These include not only the technical and operational aspects of the token system but also the behavioural, ethical, and privacy-related implications of incentivised data sharing.

Lastly, while the conceptual framework of BCR is introduced here, empirical validation of its effectiveness and performance remains a crucial task for future research. Constructing experimental setups to evaluate BCR’s performance and effectiveness in real-world applications is an essential future direction for this work.

## 6. Conclusions

This study proposed BioChainReward, a novel blockchain-based framework designed to enable the secure sharing of biomedical data with third-party entities, such as research institutions. BCR comprises four layers that leverage the advantages of blockchain technology to secure biomedical data during processing and transmission.

A key feature of BCR is its integration of smart contract technology. This innovation incorporates an AI-enabled privacy-preservation sublayer, which allows for the application of the most suitable privacy-preserving techniques based on the purpose and nature of the data request. This feature not only provides a tailored level of security for each data transaction but also ensures substantial user control over privacy settings, addressing significant concerns in the field of data security.

In addition, BCR introduces a feedback incentive mechanism, which incentivises and rewards patients for sharing their personal data, potentially increasing user participation and engagement and enriching the data available for biomedical research.

## Figures and Tables

**Table 1:** Comparison of metrics for different data-sharing approaches. In each row, the best-scoring data-sharing approach is highlighted in bold.

Metric	Centralised Databases	Federated Databases	Conventional Data-Sharing Platforms	BioChainReward
Security	Medium	Medium	Medium	**High**
Usability	**High**	**High**	**High**	**High**
Scalability	Medium	**High**	**High**	**High**
Computational complexity	**Low**	Medium	Medium	Medium
Computational cost	**Low**	Medium	Medium	Medium
Transparency	Low	Medium	Low	**High**
Data-owner control	Low	Medium	Medium	**High**
Privacy preservation	Low	Medium	Medium	**High**
Interoperability	Medium	**High**	**High**	**High**
Trust and reputation	Medium	Medium	Low	**High**
Incentive mechanism	None	None	None	**Present**
Time delay	High	Medium	High	**Low**

## Data Availability

No primary data were collected in this research.
